# Mass cytometry analysis reveals a cross-tissue immune landscape in *Actinobacillus pleuropneumoniae*-induced pneumonia

**DOI:** 10.1128/spectrum.02665-24

**Published:** 2025-04-16

**Authors:** Yanyan Tian, Xuan Jiang, Chuntong Bao, Tamin Abdelaal, Dexi Chen, Wenjing Wang, Fengyang Li, Liancheng Lei, Na Li

**Affiliations:** 1 Key Laboratory for Diagnosis and Treatment of Severe Zoonotic Infectious Diseases, Key Laboratory for Zoonosis Research of the Ministry of Education, Institute of Zoonosis, and College of Veterinary Medicine, Jilin University 623813, Changchun, China; 2 Leiden Computational Biology Center, Leiden University Medical Center 4501, Leiden, Netherlands; 3 Department of Pattern Recognition and Bioinformatics Group, Delft University of Technology 2860, Delft, Netherlands; 4 Beijing Institute of Hepatology, Beijing Youan Hospital, Capital Medical University 666122, Beijing, Beijing, China; University of Miami, Miami, Florida, USA

**Keywords:** *Actinobacillus pleuropneumoniae*, immune cell clusters, mass cytometry, immune response

## Abstract

Porcine contagious pleuropneumonia caused by *Actinobacillus pleuropneumoniae* (APP) is a fatal respiratory disease that threatens the worldwide farming industry’s health. The immune responses of extrapulmonary tissues play an important role in developing porcine contagious pleuropneumonia; however, the immune responses of extrapulmonary tissues induced by APP are rarely uncovered. Here, we used high-dimensional mass cytometry to investigate the immune cell response in the spleen and peripheral blood during APP infection in mice. We found that the immune response triggered by APP was highly tissue-specific. Numerous infection time- or tissue-specific immune cell clusters, including previously unrecognized ones, were also identified in the spleen and peripheral blood. Integrative analysis of splenic lymphoid and myeloid cell clusters maps the dynamic immune response cellular network during APP infection. Surprisingly, during the early stages of APP infection, the majority of the top 6 cell clusters contributing to the infection time-specificity in the spleen were adaptive immune cell clusters rather than innate immune cell clusters, among which CD24^hi^MHCII^+^CD8^+^T_EM_ cells exhibited a stronger expression of IFN-γ, IL-17A, and IL-10 compared to the CD24^lo^ compartment. In peripheral blood, there was unprecedented heterogeneity in the immune cell composition. Also, peripheral immune cell clusters closely related to the severity of APP infection were identified. In summary, our data provide a systemic and comprehensive overview of the immune responses to APP infection in the spleen and peripheral blood. This provides a foundation for understanding the immune pathogenesis of APP and identifying potential diagnostic biomarkers and therapeutic targets.

**IMPORTANCE:**

This study explored the cross-tissue immune dynamic landscape in the APP-induced pneumonia model by utilizing high-dimensional mass cytometry. We discovered that APP-induced immune responses are tissue-specific. Key infection-specific clusters in the spleen and peripheral blood were identified, some of which were previously unrecognized. Meanwhile, the specific functions of APP infection-related immune subsets were explored. The research systematically outlined an overview of immune responses in these tissues, deepening the understanding of APP pathogenesis and laying the foundation for the search for diagnostic and therapeutic targets.

## INTRODUCTION

*Actinobacillus pleuropneumoniae* (APP) is the causative agent of porcine contagious pleuropneumonia, a highly transmissible respiratory disease with a significant fatality rate (1). Due to the lack of vaccines with cross-immune protection between different serotypes of APP, the mixed infection with swine influenza, swine fever, swine pneumonic disease, and the growing concerns about antibiotic residues and bacterial resistance, APP infection has resulted in huge losses for the pig farming industry (2). Therefore, it is critical to identify new targets for the prevention and treatment of porcine-contagious pleuropneumonia.

Pathogen-induced immune responses determine the development of pneumonia, so understanding the composition and characteristics of immune cell subsets during APP infection is essential. While several immune cell subsets have been reported in APP infection, the majority of research has been focused on the lung tissue (3, 4). It is worth noting that the immune response of extrapulmonary tissues also plays an important role in the process of APP infection, especially in the spleen and peripheral blood. Different microenvironments endow immune cell subsets with unique immune characteristics and functions (5). Proteomics analysis of alveolar lavage fluid, peripheral blood mononuclear cells (PBMCs), and serum from APP-infected piglets revealed that the immune response in PBMCs and serum was faster and lasted longer than that in the lungs, indicating that the immune response in the lungs significantly differed from that in the peripheral blood (6). In addition, the spleen is required for the development of T cells, the production of inflammatory cytokines, and the clearance of bacteria during bacterial infection (7). Therefore, a comprehensive understanding of the composition and characteristics of the immune cells in extrapulmonary tissues is crucial for APP infection, which may open a novel avenue for the prevention and treatment of APP infection.

Given the important role of immune cells and immune responses in bacterial pneumonia, in-depth characterization of immune cell composition and characteristics in extrapulmonary tissues could better understand the immunopathogenic mechanism of APP infection. In this study, we applied mass cytometry to thoroughly analyze the characteristics and makeup of immune cells in mouse spleen and peripheral blood during APP infection. We discovered immune cell clusters with tissue specificity and infection stage specificity, generating a dynamic immune landscape during APP infection, which advanced our knowledge of APP pathogenesis.

## MATERIALS AND METHODS

### Bacterial strains and conditions

APP serotype 5 reference strain L20 (APP 5b L20) and APP serotype 1 reference strain (APP CVCC259) were obtained from the Shanghai Entry-Exit Inspection and Quarantine Bureau (Shanghai, China) and cultured according to the previous method (3). Briefly, single colonies were transferred to 3 mL of Brain Heart Infusion (BHI, BD) liquid medium containing 10% heat-inactivated horse serum (Kangyuan Biologicals) and 15 µg/mL nicotinamide adenine dinucleotide (NAD, Sigma), and cultured for 6 hours (h) at 37°C and 180 rpm/min agitation using a spectrophotometer adjusted by optical density (OD), and finally centrifuged at 3,500 × RCF(*g*). The bacteria were washed three times with phosphate-buffered saline (PBS).

### Experimental infection

18–22 g, 6- to 8-week-old female ICR mice were purchased from Jilin University Laboratory Animal Centre. All animal studies were conducted by the National Guidelines for the Welfare of Laboratory Animals (Ministry of Science and Technology of China, 2006) and approved by the Animal Welfare and Research Ethics Committee of Jilin University (License No. 201710034). The mouse APP infection model was constructed according to previous methods (3). APP 5b L20-infected group (6.5 × 107 CFU in 30 µL of sterile saline injected nasally per mouse), APP CVCC259-infected group (6.5 × 107 CFU in 30 µL of sterile saline injected nasally per mouse), and control group (30 µL of sterile saline injected nasally per mouse). Mice in the control group were anesthetised before infection, weighed, and then executed. The body weights of the mice were observed at 6 h, 12 h, 24 h, 48 h, 7 d, and 14 d after infection (as shown in each experiment), and the mice in each group were anesthetised and executed, and tissues (spleens, inguinal lymph nodes) were collected for further analysis.

### Histological analysis

The lungs and spleens of the mice were fixed in 10% buffered formalin. After paraffin embedding, the paraffin block was cut into 5 µm using a microtome, first deparaffinized and rehydrated, and then the stained part was stained with eosin for 3 min, dehydrated in graded alcohols, soaked in xylene, naturally dried in a fume cupboard, and then mounted with balsam. Finally, slides were observed and scanned with a PANNORAMIC MIDI II automatic digital slide scanner (3DHISTECH, Budapest, Hungary).

### Cell isolation

The spleens and inguinal lymph nodes were cut into small pieces, harvested by mechanical grinding on a 70 µm Nylon filter in a 50 mL centrifuge tube, and lysed using Red Blood Cell Lysis Buffer (Solarbio, Beijing, China) to remove the red blood cells. To obtain enough blood cells, the blood samples within each time point were collected in an anticoagulation tube as one sample. The blood was then lysed with Red Blood Cell Lysis Buffer (Solarbio, Beijing, China) to obtain white blood cells. Resuspend all isolated cells in the staining buffer to obtain a single-cell suspension, and store samples on ice until mass cytometry antibodies are added.

### Mass cytometry antibody staining and data acquisition

Mass cytometry antibody staining and data acquisition were performed according to our previous methodology (3). Briefly, antibodies were coupled to the corresponding metal tags using the MaxPar X8 Antibody Labelling Kit (Fludigm Science). First, to identify dead cells, single-cell suspensions from different tissues were incubated with 0.5 mL of 2 µM Cell-ID cisplatin. Next, cell suspensions from different tissues were mixed well with the antibody mixture and incubated on ice for 30 min. After staining, cells were stained with 0.5 mL 125 nM Cell-ID intercalator-Ir (Fludigm Science) to label all the cells in the Fix and Perm Buffer (Fludigm Science) overnight at 4°C. Finally, cells were acquired with the Helios mass cytometer, and data were normalized using EQ Four Element Calibration Beads (Fludigm Science).

### Mass cytometry data analysis

The analysis of mass cytometry data were performed as reported in our previous study (3). Briefly, data were collected and normalized, and samples were pre-processed using Flowjo (version 10.4) to obtain single live CD45^+^ cells for the study. Next, cells from different samples were analyzed using Cytoplore^+H-SNE^ software as previously reported (8). Briefly, the t-SNE dimensionality reduction algorithm is applied to various tissues to visualize high-dimensional data. The default setting (Perplexity: 30; iteration: 1,000) was carried out individually within each tissue. All H-SNE, t-SNE plots, and Gaussian Mean shift clustering-derived cell clusters were generated by the Cytoplore software. Each cell cluster contained at least 100 cells. In Matlab 2015, hierarchical clustering of phenotypic heatmaps was used using Euclidean correlation and average linkage clustering methods (9). For the cluster t-SNE maps, the data matrix of the clusters of CD45^+^ cells in a single sample was normalized and calculated, and the top 10 principal components with the highest variance were selected as inputs to the t-SNE analysis to cluster clusters with similar characteristics together. Histograms were produced using GraphPad Prism 8 software.

### Flow cytometry

Single-cell suspensions of mouse immune cells were obtained according to the method of cell separation described above. For cell surface antigen staining, cells were stained with the following fluorochrome-conjugated antibodies, BV510-anti-mouse/human CD11b (M1/70, Biolegend), BV421-anti-mouse/human CD11b (M1/70, Biolegend), PE-anti-mouse F4/80 (W20065B, Biolegend), BV605-anti-mouse F4/80 (W20065B, Biolegend), PerCP/Cy5.5-anti-mouse CD3 (17A2, Biolegend), APC-anti-mouse CD3 (17A2, Biolegend), APC-anti-mouse CD4 (GK1.5, Biolegend), BV421-anti-mouse CD4 (GK1.5, Biolegend), BV510-anti-mouse CD4 (GK1.5, Biolegend), PE/Dazzlem-anti-mouse CD8a (53–6.7, Biolegend), PE/Cyanine7-anti-mouse CD8 (53–6.7, Biolegend), BV605-anti-mouse CD8a (53–6.7, Biolegend), BV510-anti-mouse Ly6C (HK1.4, Biolegend), BV421-anti-mouse MHCII (M5/114.15.2, Biolegend), APC/Cy7-anti-mouse CD62L (MEL-14, Biolegend), PE/Dazzlem-anti-mouse CD62L (MEL-14, Biolegend), FITC-anti-mouse CD44 (IM7, Biolegend), PE/Cyanine7-anti-mouse CD24 (30-F1, Biolegend), PerCP/Cy5.5-anti-mouse CD11c (N418, Biolegend), APC/Cy7-anti-mouse CD11c (N418, Biolegend), PE-anti-mouse Ly6G (1A8, Biolegend), PerCP-eFluor 710-anti-mouse Ly6G (1A8, Biolegend), and PE-anti-mouse TLR4 (SA15-21, Biolegend). For intracellular cytokine staining, spleen single cells were stimulated with Phorbol 12-myristate 13-acetate (PMA) (50 ng/mL, Sigma) and ionomycin (1 mg/mL, Sigma) for 8 h at 37°C, and 1× brefeldin A and monensin solution (BioLegend) were added for the final 4 h. Cultured cells were washed twice using FACS buffer and stained with surface markers. The cells were then fixed, permeabilized with Cyto-Fast Fix/Perm Buffer Set (BioLegend) and incubated with intracellular antibodies for 30 min at 4°C, PE-anti-mouse IL-10 (JES5-16E3, Biolegend), APC/Cy7-anti-mouse IL-17A (TC11-18H10.1, Biolegend), and APC-anti-mouse IFN-γ (XMG1.2, Biolegend). Cells were analyzed on a flow cytometer (Beckman, USA), and data were analyzed with FlowJo (version 10.4). The acquired data were analyzed using FlowJo software. Through the gating strategy, corresponding cell populations were delineated on the scatter plot based on the fluorescence signal characteristics of the cells, and the software could automatically calculate the absolute numbers of each cell population.

### Statistical analysis

After the normality test, multiple group comparisons were made using the Kruskal-Wallis test and Dunn test. Mann-Whitney test was used for two group comparisons. *P ≤ 0.05, **P ≤ 0.01, and ***P ≤ 0.001.

## RESULTS

### APP-induced immune responses are tissue-specific

**Fig 1:**
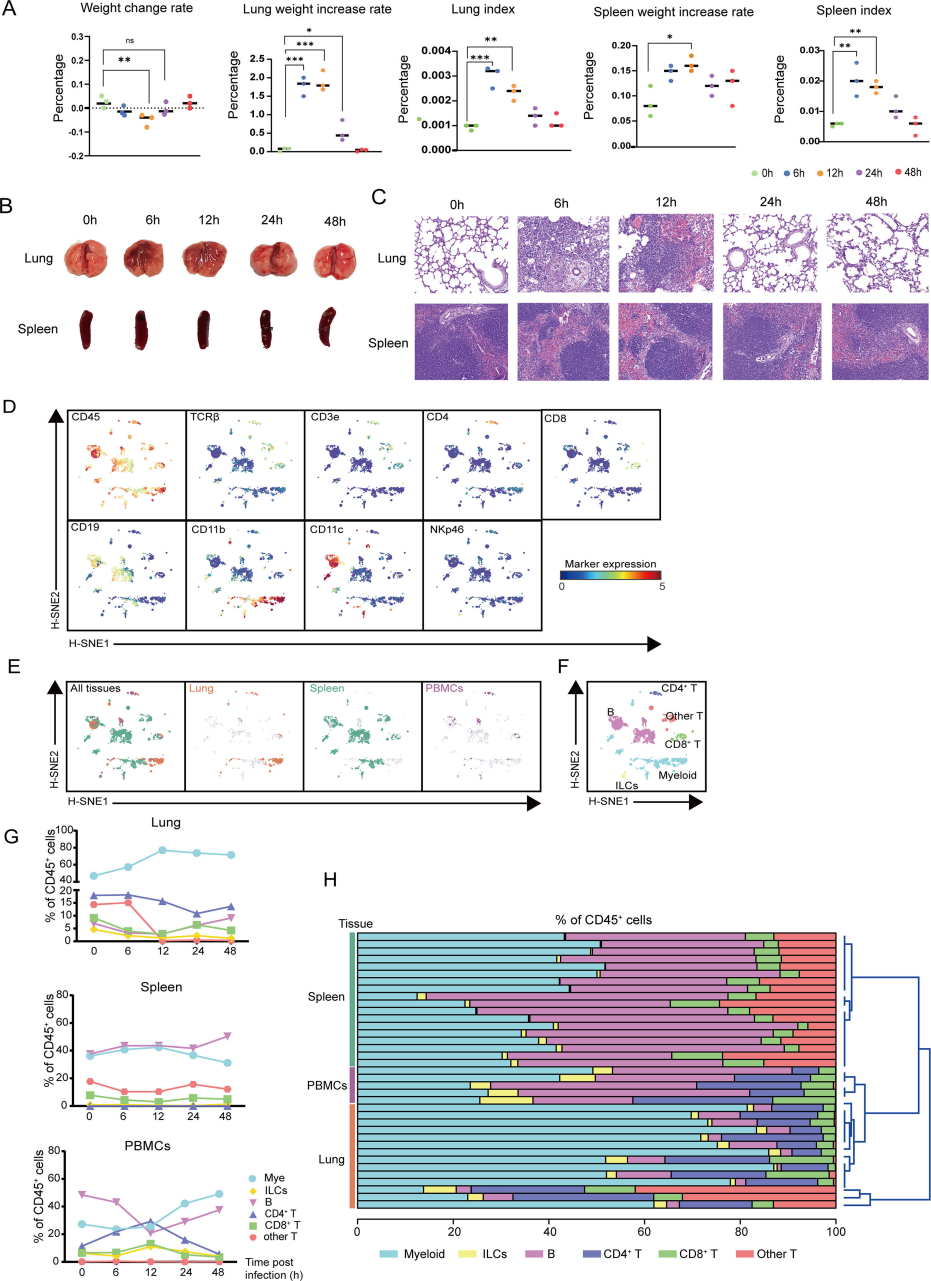
The immune responses against APP infection are tissue-specific. (**A, B**) Changes in lung weight change rate, lung weight increase rate, lung index, spleen weight increase rate, spleen index, and pathological changes at 6, 12, 24, and 48 h after APP infection compared to no infection (0 h). (**C**) HE staining was used to observe pathological damage in the lung and spleen at 6, 12, 24, and 48 h after APP infection compared to no infection (0 h) (20×). (**D**) HSNE embeddings of 1,290,940 immune cells derived from murine lung (*N* = 14), spleen (*N* = 18), and PBMCs (*N* = 5) at the overview level. Each dot represents a landmark, whose size is proportional to the number of cells it represents. Colors indicate the ArcSinh5-transformed expression value of each indicated marker. (**E**) HSNE plots show the tissue origin in different colors. (**F**) HSNE plots show the major immune lineage cluster partitions in different colors. (**G**) Cell frequencies of each major immune lineage in CD45^+^ cells across three different tissues during APP infection. (**H**) The stacked bar graph shows the cell frequencies of the major immune lineages (as % of CD45^+^ immune cells) in each sample and hierarchical clustering.

We first established a mouse model of APP infection as previously reported (3). The weight change rate of mice dramatically decreased until 24 h after APP infection, and the lung weight increase rate and the lung index increased at 6 h and 12 h and decreased at 24 h and 48 h, suggesting that mice recovered from the infection after 12 h. Spleen index and spleen increase rate increased at 6 h and 12 h (Fig. 1A). Similar results were obtained on the lung and spleen pathological lesions (Fig. 1B). After 6 h and 12 h of APP infection, it caused purulent alveolar pneumonia, neutrophils filled the alveolar cavity, accompanied by fibrinous exudation, edema, hemorrhage, and local necrosis, which gradually recovered in 24 h and 48 h. After 6 h and 12 h of APP infection, the spleen was congested and swollen, and the red pulp widened and gradually recovered at 24 h and 48 h (Fig. 1C). We applied a previously described mass cytometric panel consisting of 26 metal-labeled antibodies (3), which included antibodies for immune profiling, cell differentiation, activation, adhesion, and APP-specific recognition to probe the phenotypic and quantitative complexity of mouse immune cells. The single live CD45^+^ immune cells were distinguished by event length, center, residual, and width parameters, DNA staining, and CD45 antibody staining (10). To determine the immune response profiles among tissues, we pooled all the data (1,290,940 CD45^+^ cells) derived from 14 lungs (219,967 cells), 18 spleens (913,382 cells), and 5 blood samples (157,951 cells) and carried out a 5-level HSNE analysis in Cytosplore (8). Here, the landmarks described the immune composition among all three tissues (Fig. 1D). Strikingly, the global differences in immune response in different tissues were displayed by visualizing the tissue origin of the cells (Fig. 1E). Next, we determined the major immune lineages based on marker expression profiles (Fig. 1D), which identified CD3^−^CD19^−^NKp46^−^CD11b^+^/CD11c^+^ myeloid cells, CD3^−^CD19^−^CD11b^−^CD11c^−^ innate lymphoid cells (ILCs), CD3^+^TCRβ^+^CD4^+^ T cells, CD3^+^TCRβ^+^CD8^+^ T cells, CD3^+^TCRβ^+^CD4^-^CD8^-^ (other) T cells, and CD19^+^ B cells (Fig. 1F). We then quantified the percentage of the major lineage within each tissue and found that compared with blood myeloid cells were more abundant in the lung and spleen without infection, while these cells gradually increased in lung and blood and decreased in the spleen after infection (Fig. 1G). As expected, B cells were the most profound population in the spleen and blood, which increased in the spleen during infection while the frequencies of these cells reached the lowest point in the blood 12 h after infection (Fig. 1G). Compared to the blood, the “other T cells” were relatively more abundant in the lung and spleen, and dramatically decreased in the lung 12 h after infection (Fig. 1G). Importantly, the frequencies of all the immune lineages except “other T cells” changed substantially 12 h post-infection in the blood but not in other tissues (Fig. 1G). Moreover, unbiased hierarchical clustering of cell frequencies grouped the samples in a tissue-specific manner (Fig. 1H). Together, the global analysis revealed that the immune responses against APP infection were tissue-specific.

### Characteristics of the myeloid cell response in the spleen post-APP infection

**Fig 2:**
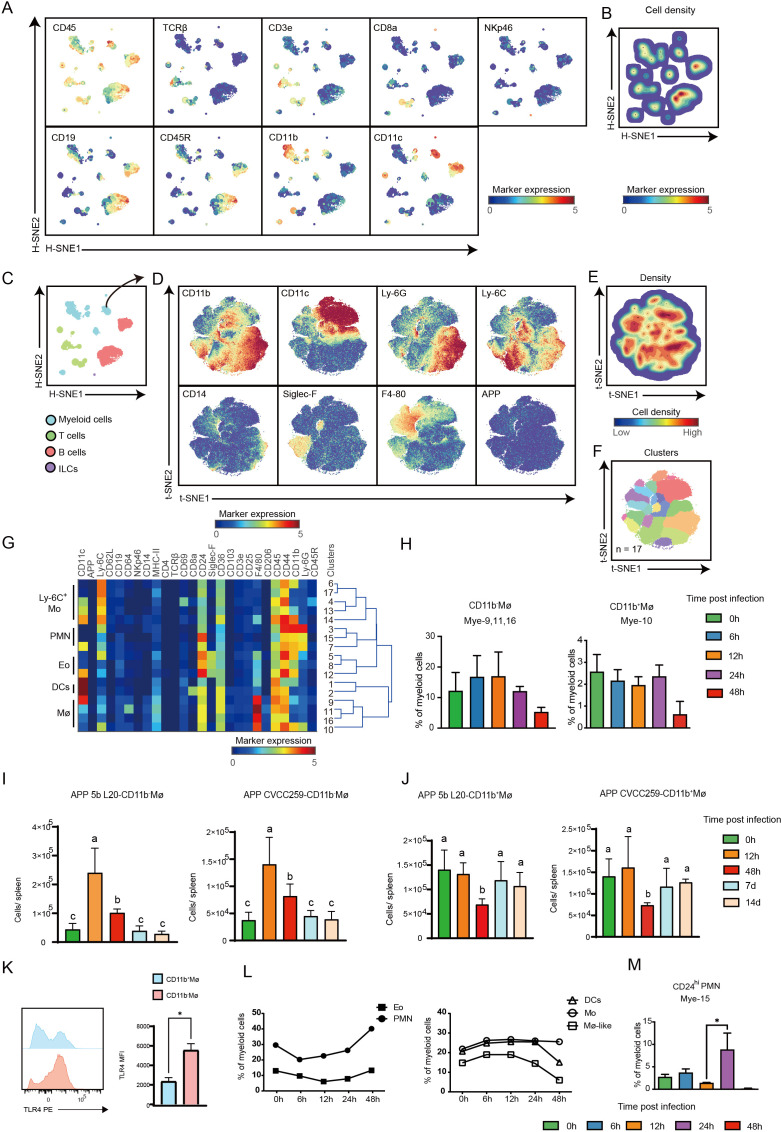
Cluster identification in the myeloid cell compartment in the spleen. (A) The HSNE embeddings of 913,382 immune cells derived from the spleen (*N* = 18). Each dot represents a landmark, whose size is proportional to the number of cells it represents. Colors indicate the ArcSinh5-transformed expression value of each indicated marker. (**B**) The HSNE plot shows the cell density. (**C**) A HSNE embedding of 913,382 immune cells derived from the spleen (*N* = 18). Colors represent different major immune lineages. (**D**) t-SNE embeddings of 253,778 myeloid cells showing the ArcSinh5-transformed expression value of each indicated marker. (**E**) A density map showing the local probability density of the embedded cells. (**F**) A t-SNE plot showing cluster partitions. (**G**) Heatmap displaying the median marker expression value and hierarchical clustering of the markers for 17 clusters identified in panel F. (**H**) Cell frequency of Mye-9,11,16,10 in myeloid cells in the spleen. (**I, J**) Quantification of CD11b^+^ Mø, CD11b^−^Mø after infection with APP 5b L20 and APP CVCC259. Error bars indicated mean ± SD. (**K**) The expression levels of TLR4 on CD11b^−^Mø and CD11b^+^Mø in the spleen at 12 h after infection with APP 5b L20 using flow cytometry. (**L**) Cell frequencies of indicated immune clusters in myeloid cells in the spleen. (**M**) Cell frequency of Mye-15 in myeloid cells in the spleen.

As the largest peripheral immune organ, the spleen index increased from 0 to 12 h and gradually decreased from 24 to 48 h after APP infection, suggesting that the mice were severely ill at 6 h and 12 h and recovered from the infection after 12 h (3). We next performed H-SNE dimensionality reduction analysis of all CD45^+^ immune cell populations (913,382 cells) in the spleen and identified four major immune lineages, namely myeloid cells, T cells, B cells, and ILCs, based on marker expression profiles and cell density characteristics (Fig. 2A through C). We used Gaussian mean-shift clustering for further dimensionality reduction and clustering analysis of myeloid cells and identified 17 phenotypically distinct myeloid cell clusters that clustered into five groups, namely Ly-6C^+^ monocytes (Mo), polymorphonuclear neutrophils (PMN), eosinophils (Eo), dendritic cells (DCs), and macrophages (Mø) (Fig. 2C through G). Interestingly, the majority of Mφ (mye-9,11,16) in the spleen did not express CD11b, possibly representing mature macrophages in the spleen (11, 12), and its percentage was elevated at 6–12 h and decreased at 24 h and 48 h of infection (Fig. 2H). CD11b^+^Mø (mye-10), on the other hand, decreased at 48 h post-infection (Fig. 2H). The cell number of CD11b^−^Mø and CD11b^+^Mø per spleen was further determined at longer times post-infection using flow cytometry. CD11b^-^Mø counts were significantly elevated at 12 h and returned to the uninfected level at 7–14 d (Fig. S1; Fig. 2I). Consistent with the mass cytometric data, CD11b^+^Mø counts were significantly reduced at 48 h post-infection and also returned to the uninfected level at 7–14 d (Fig. S1; Fig. 2J). Similar results were obtained in mouse spleens post-APP CVCC259 infection (Fig. S1; Fig. 2I through J). Compared with CD11b^+^Mø, CD11b^-^Mø expressed a higher level of TLR4, suggesting that CD11b^−^Mø may have a strong ability to resist infection (Fig. S1; Fig. 2K). Unlike the myeloid cell composition in the lung (3), Eo was the most dominant population, which first decreased and then increased during infection (Fig. 2L). Interestingly, compared with the lung (3), PMN in the spleen showed less heterogeneity and decreased at 6–12 h and increased at 24 h and 48 h (Fig. 2L). However, only the CD24^hi^ PMN, exhibiting the characteristics of apoptotic cells, was significantly enriched at 24 h after APP infection (Fig. 2M). Contrary to the PMN, the percentage of DCs and Mø increased at 6–12 h and decreased at 24 h and 48 h, whereas Mo increased at 6 h and maintained a high level until 48 h (Fig. 2L). Altogether, our data reveal the composition and characteristics of myeloid cell subsets in the spleen during APP infection.

### Characteristics of the lymphoid cell response in the spleen post-APP infection

**Fig 3:**
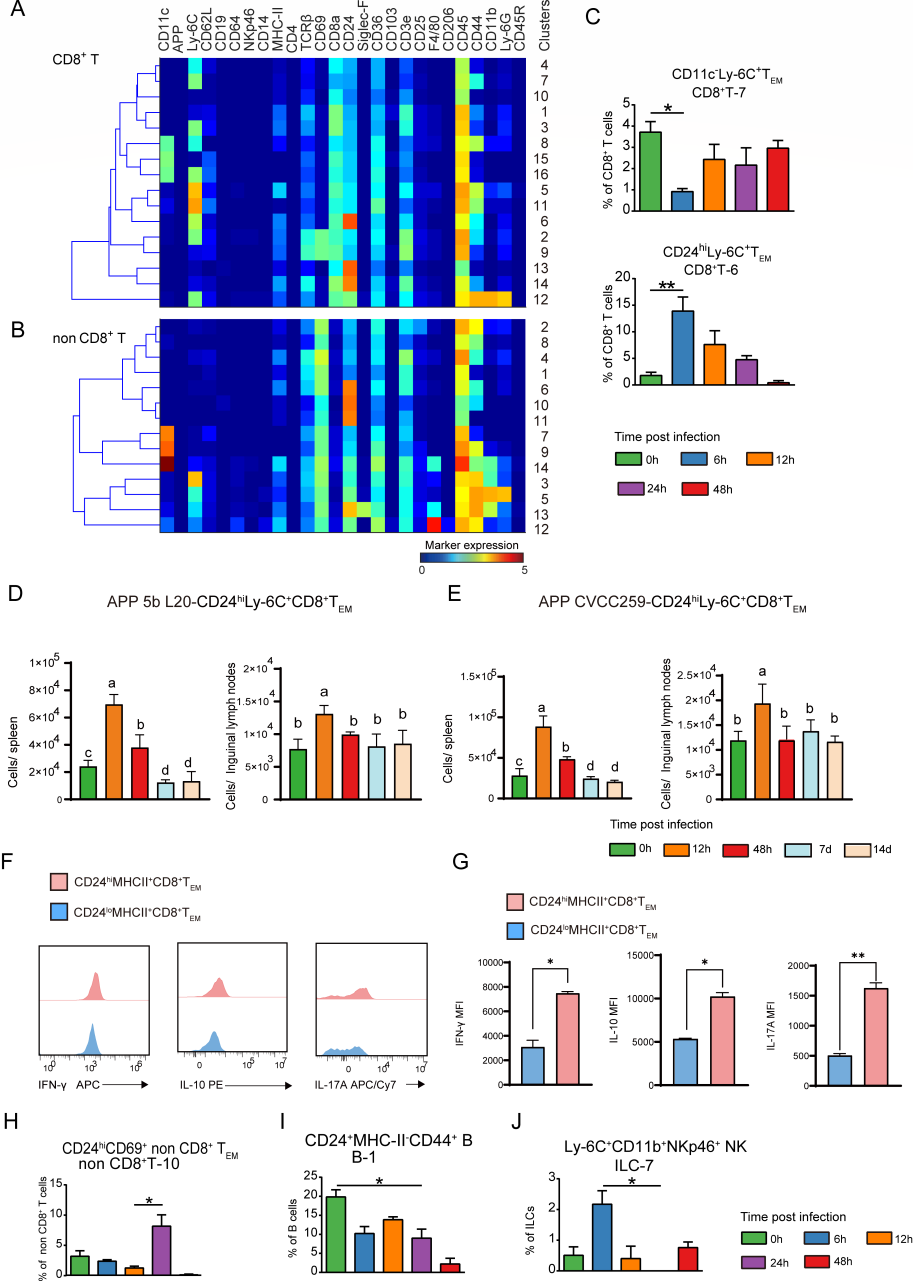
Cluster identification in the lymphoid cell compartment in the spleen. (**A, B**) Heatmaps display the median marker expression value and hierarchical clustering of the markers for clusters identified in each major immune lineage. (**C**) Cell frequencies of the indicated immune clusters within each major immune lineage in the spleen. (**D, E**) Quantification of CD24^hi^Ly-6C^+^CD8^+^T_EM_ after infection with APP 5b L20 and APP CVCC259. Error bars indicated mean ± SD. (**F**) Flow cytometry was used to detect the expression levels of IFN-γ, IL-17A, and IL-10 in CD24^hi^MHCII^+^CD8^+^T_EM_ and CD24^lo^MHCII^+^CD8^+^T_EM_ after stimulation with PMA and ionomycin. (**G**) Levels of cytokines (IL-17A, IL-10, and IFN-γ) secreted by CD24^hi^MHCII^+^CD8^+^T_EM_ and CD24^lo^MHCII^+^CD8^+^T_EM_ were detected by flow cytometry (error bars represent median ±SD). (**H–J**) Cell frequencies of the indicated immune clusters within each major immune lineage in the spleen.

Similarly, based on the marker expression profiles, we identified 48 phenotypically unique clusters for the lymphoid populations (16 CD8^+^ T, 14 non-CD8^+^ T, 10 ILC, and 8 B-cell clusters) (Fig. 3A and B; Fig. S2). At 6 h post-infection, there was a considerable reduction in the percentage of CD11c^-^Ly-6C^+^CD8^+^ T_EM_ cells. On the contrary, CD24^hi^Ly-6C^+^CD8^+^ T_EM_ cells were significantly increased at 6 h of the infection, and their fraction progressively dropped as the infection time prolonged (Fig. 3C). Moreover, the absolute number of CD24^hi^Ly-6C^+^CD8^+^T_EM_ cells per spleen increased significantly after 12 h of infection and significantly decreased at 7–14 d. In the inguinal lymph nodes, these CD24^hi^Ly-6C^+^CD8^+^T_EM_ cells also showed a significant increase at 12 h, with their numbers returning to the uninfected level at 48 h (Fig. 3D; Fig. S3). Similar results were observed in APP CVCC259-infected mice (Fig. 3E; Fig. S3). In addition, compared to CD24^lo^MHCII^+^CD8^+^T_EM_ cells, CD24^hi^MHCII^+^CD8^+^T_EM_ cells exhibited a stronger capacity to produce cytokines, including IFN-γ, IL-17A, and IL-10 (Fig. 3F and G; Fig. S3). Remarkably, CD69, a tissue-resident or activation T-cell marker, was expressed by all CD8^-^ T cells in the spleen (Fig. 3B). 24 h after infection, CD24^hi^CD69^+^CD8^-^ T_EM_ cells were more abundant in the spleen (Fig. 3H), indicating that the CD24^hi^CD8^-^ T_EM_ cell subsets might be involved in the suppression of the inflammatory response at the recovery stage of APP infection.

Consistent with previous studies, there were a large number of B lymphocytes in the spleen, which gradually increased with the progression of infection (Fig. 3H). Based on the differential expression of markers such as CD11c, Ly-6C, MHC-II, and F4/80, these B lymphocytes were further divided into 9 cell populations (Fig. S3A). Among them, there was a notable decrease in the percentage of CD24^+^MHC-II^-^CD44^+^ B cells (Fig. 3I). In addition, all ILCs in the spleen were NK cells, among which Ly6C^+^CD11b^+^ NK cells were enriched in the early stage (6 h) of APP infection (Fig. 3J; Fig. S2B). Altogether, our data built the landscape of lymphoid cell clusters in the spleen post-APP infection and identified the infection-phase-specific clusters.

### Integrated analysis of the entire immune system reveals the infection-associated networks of immune clusters in the spleen

**Fig 4:**
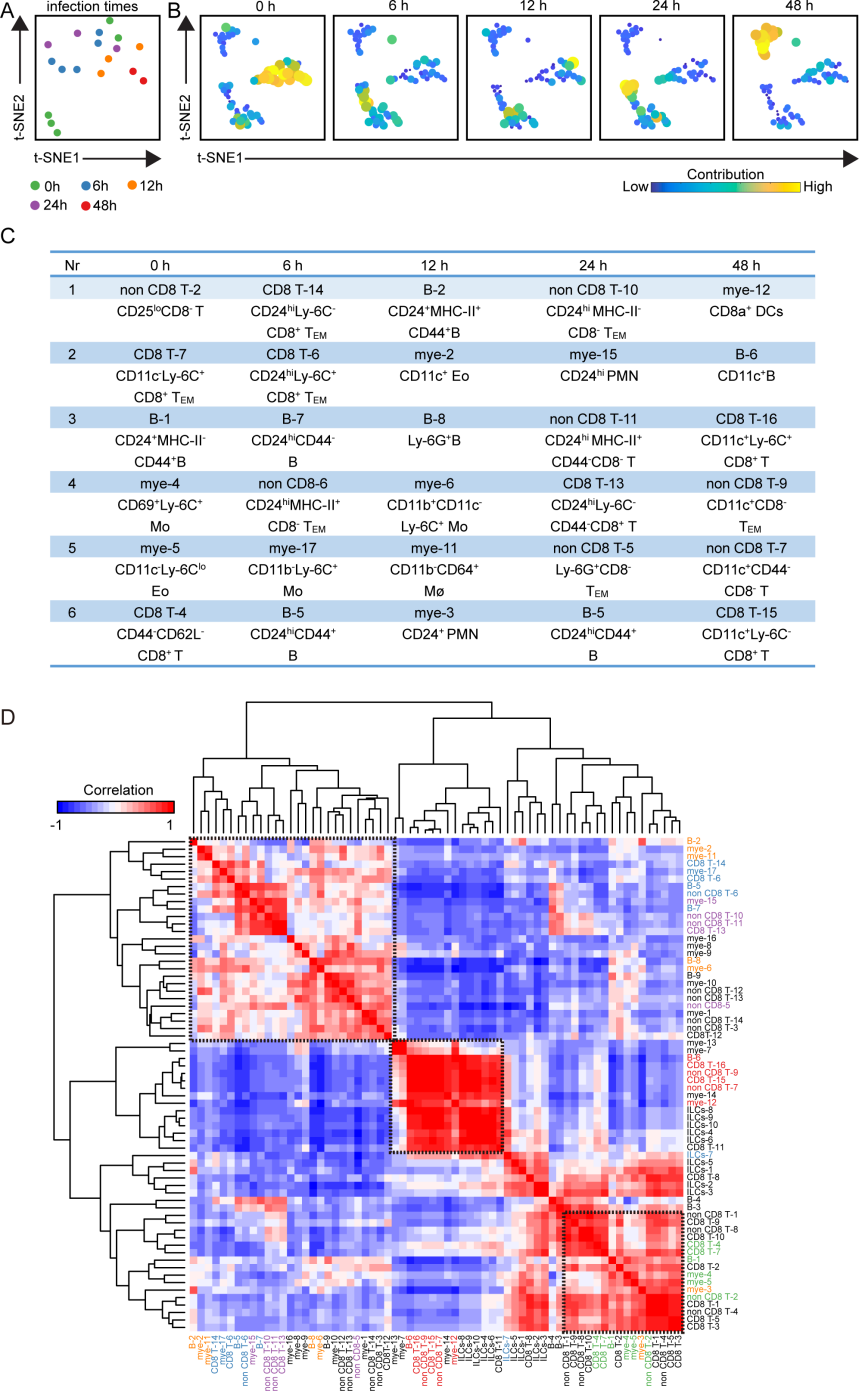
Infection-associated clusters are revealed in the spleen. (A) A t-SNE embedding of 18 spleen samples, where the t-SNE was computed based on the cell frequencies of 65 immune clusters (% of CD45^+^ cells). One dot represents one sample. (**B**) t-SNE embeddings of 65 immune clusters from 18 samples. One dot represents one cluster. The size of the dot is proportional to the cell frequency value. The more similar the cell frequencies are across tissues, the closer the clusters are. (**C**) A table depicts the top six clusters contributing to the infection time-specific t-SNE signatures. (**D**) A heatmap shows a correlation among 65 immune clusters based on the cell frequencies of total CD45^+^ cells in each sample and hierarchical clustering. The top six clusters and the clusters significantly differentially enriched in different infection times are highlighted in different colors. Green: 0 h, yellow: 6 h, blue: 12 h, purple: 24 h, and red: 48 h.

**Fig 5:**
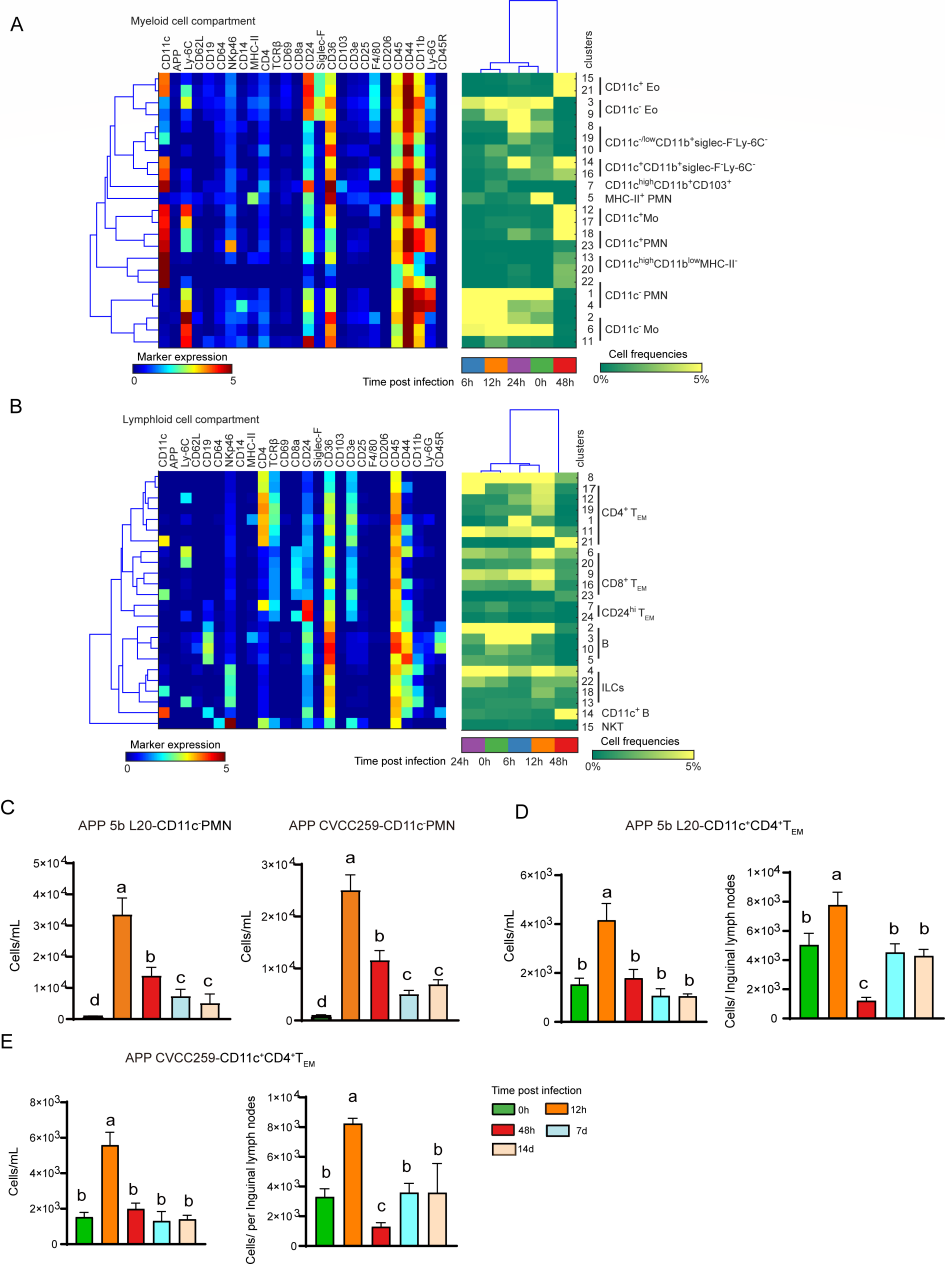
Mass cytometric analysis reveals the immune composition of peripheral blood after APP infection. (**A-B**) Heatmap (blue-to-red scale) displaying the median marker expression value and hierarchical clustering of the markers for myeloid and lymphoid cell clusters (left panel). Heatmap (green-to-yellow scale) showing the corresponding cell frequencies of each cluster within the myeloid or lymphoid cell compartment at each time point. The dendrogram shows the hierarchical clustering of samples in different infection times (right panel). (**C**) Quantification of CD11c^-^PMN in peripheral blood after infection with APP 5b L20 and APP CVCC259. Error bars indicated mean ± SD. (**D, E**) Quantification of CD11c^+^CD4^+^T_EM_ in peripheral blood and inguinal lymph nodes after infection with APP 5b L20 and APP CVCC259. Error bars indicated mean ± SD.

To reveal the immune cell network in the spleen after APP infection, we integrated all the immune clusters and performed a t-SNE analysis based on the cell cluster frequencies. There were no clear infection-related patterns among the samples except at 48 h (Fig. 4A). Next, the top six cell clusters contributing to the infection time-specific t-SNE signatures were identified (Fig. 4A and B). Surprisingly, at the early stage of infection, the majority of these top contributors were adaptive immune cell clusters (70%, 21/30) rather than innate immune cell clusters (30%, 9/30) (Fig. 4C). Moreover, the numbers of T-cell clusters exceeded those of B cells (Fig. 4C). In this context, expected clusters were identified in the spleen such as Ly-6C^+^ Mo, CD64^+^ Mø, and CD24^+^ PMN. In addition, several previously unrecognized clusters associated with APP infection were identified, including Eo, Ly-6G^+^ B, and several CD24^hi^ T-cell clusters (Fig. 4C). Next, a correlation analysis was performed on all the clusters, revealing that the samples from 48 h and part from 0 h displayed a clear infection-related pattern, while the others were not, consistent with the findings in Fig. 5A (Fig. 4D). Together, the integrated system-wide analysis maps the immune response cellular network during APP infection in the spleen and indicates that adaptive immune clusters play a key role in the early stage of bacterial infection.

### Identification of infection-associated clusters in peripheral blood

To explore the immune cell composition in peripheral blood during APP infection, we performed a similar mass cytometry analysis and identified 22 myeloid and 24 lymphoid cell clusters (Fig. 5; Fig. S4). In the myeloid cell compartment, known cell subsets such as Eo, PMN, and Mo were identified. In addition, several previously unrecognized clusters (Mye-8, 19, 10, 14, 16) were also identified. Interestingly, MHC-II expressing PMN were also detected in peripheral blood at 0 h, indicating the presence of PMN cells with antigen-presenting cell characteristics (Fig. 5A). After infection, the number of CD11c^-−^ PMN and CD11c^−^ Mo increased at 6–12 h, decreased at 24 h, while at 48 h CD11c expression was uniform on myeloid cells (Fig. 5A). Consistent with the mass cytometric data, flow cytometric assays also showed that the number of CD11c^−^ PMN increased at 12 h and then decreased along the infection time until day 7 (Fig. 5C; Fig. S5A and B). Moreover, Siglec-F^+^ Eo was either CD11c^−^ or CD11c^+^ Eo, where the former was present in 0–24 h samples, while the latter was found in 48 h samples (Fig. 5A). In the lymphoid compartment, the majority of cell clusters were CD4^+^ T_EM_ and CD8^+^ T_EM_ cells in which the CD11c^+^CD4^+^ T_EM_ cell cluster (lym-21) was dominant 48 h after infection while the other T-cell clusters were enriched 0–24 h (Fig. 5B). Further analysis using flow cytometry found that the cell numbers of CD11c^+^CD4^+^T_EM_ per milliliter increased at 12 h and returned to baseline levels at 48 h post-infection. In the inguinal lymph nodes, these cells were significantly reduced at 48 h and increased to the uninfected levels at day 7 and day 14 (Fig. 5D; Fig. S5 C and D). Similar results were obtained post-APP CVCC259 infection (Fig. 5D and E; Fig. S5C and D). Five B-cell clusters were identified, and MHC-II^+^CD45R^+^ B cells (lym-3) increased at 6 h and decreased from 12 to 48 h after infection (Fig. 5B). Meanwhile, a B-cell cluster expressing CD11c (lym-14) was identified in 48 h samples. ILCs, mainly NK cells, were divided into four clusters based on the differential expression of CD11c, Ly-6C, and CD11b (Fig. 5B). After the unbiased hierarchical clustering, the samples from 6 h and 12 h were grouped, whereas those from 0 h and 24 h clustered together, indicating that the myeloid and lymphoid cell composition and status of 0 h and 6 h samples were similar to that of 24 h and 12 h samples, respectively (Fig. 5). Finally, the immune composition at 24 h was compatible with the recovery stage after APP infection, which was consistent with the findings in the lung and indicated that the immune composition in peripheral blood may reflect the infection status in the lung. Thus, the infection-associated immune cell clusters were readily identified in peripheral blood. The composition of these immune cells can be used to determine the progress of APP infection.

## DISCUSSION

Porcine-contagious pleuropneumonia caused by *Actinobacillus pleuropneumoniae* (APP) is a severe and contagious respiratory disease, leading to large financial losses to the pig industry worldwide (13). Since prevention and treatment for APP infection are now exceedingly challenging, a detailed understanding of immune cell dynamics during pneumonia holds the potential to uncover new strategies. Although APP is not a zoonotic bacterium, it causes similar pathological damage to that seen in *S. aureus* infection, including damage to alveolar epithelial cells and necrotic detachment of alveolar walls. APP infection is also associated with a significant increase in the recruitment and activation of neutrophils in lung tissues, as well as the release of various inflammatory factors. Similarly, *Klebsiella pneumoniae* infection triggers the recruitment and activation of a large number of inflammatory cells (14–16). In addition, APP anti-infective targets are equally applicable to pneumococcal and Staphylococcus aureus infections, and the immune responses induced by APP could provide a basis for other bacterial pneumonia studies (17). Our group previously established and compared APP-infected mouse and piglet models and found that the immune cell changes observed in ICR mice after APP infection were similar to those in APP-infected piglets (3). This suggests that the mouse models can partially explain the immune response in piglets following APP infection. However, there is currently no comprehensive analysis targeting APP infection that simultaneously interrogates the innate and adaptive immune subsets across various tissues, particularly in the spleen and peripheral blood. Furthermore, we built the immune atlas in the spleen and peripheral blood, where immune cell clusters, including previously unrecognized ones that mark the infection stage and tissues, were identified. In addition, immune cell clusters in peripheral blood closely related to the severity of APP infection were readily identified. In addition, we selected the key immune clusters identified in APP serotype 5 infection to track their changes in APP serotype 1 infection mice. Similar data were obtained between these two serotypes, suggesting the generalizability across multiple serotypes (18). In addition, the key immune cell subpopulations were further dissected in the spleen, peripheral blood, and inguinal lymph nodes at 0 h, 12 h, 48 h, 7 d, and 14 d post-infection, allowing us to identify the early markers for different infection stages. Thus, our study, to our knowledge, was the first one to systematically dissect the immune response in the extra lung tissues post-APP infection, providing resources for the immune pathogenesis, potential diagnostic markers, and therapeutic targets for bacterial pneumonia.

With a number of red marrow and marginal zone (MZ) macrophages for removing pathogens from the bloodstream, the spleen is an important organ in the regulation of disseminated microorganisms (19). Also, the vital role of the spleen in bacterial pneumonia is receiving increasing attention. It has been reported that PMN in the spleen are efficient in removing the damage produced by *pneumococci* (20). PMN are direct immune effector cells of the antimicrobial response and are rapidly recruited to the infection site after infection. Our previous results showed that PMN in the spleen rapidly recruited to the lungs in the early stage of APP infection to exert an anti-infective effect (3). In addition to this, we found that CD24^hi^PMN increased significantly after 24 h of APP infection in the spleen. CD24 cross-linking could significantly accelerate PMN death in a cysteine-dependent manner (21). This suggests that PMN are in an apoptotic state during the recovery phase of APP infection, thus avoiding excessive inflammation. It has been shown that bacterial infection leads to a rapid infiltration of Eo into the site of infection, accompanied by a systemic increase in large amounts of Eo as well as elevated levels of alpha-defensin, which exerts an anti-infective effect (22). Our results also showed that Eo in the spleen decreases during 6–12 h of APP infection, a period of severe injury in the spleen. On the contrary, the number of Eo in the spleen rebounded during the recovery period of APP infection (24–48 h), suggesting Eo may have a protective role in APP infection. Hee et al. demonstrated that in psoriasis, Eo promotes PMN infiltration into the inflammatory milieu (23), which could explain the synchronization of Eo and PMN changes in the spleen after APP infection in our results. Ly6C^+^ Mø were accumulated in the mouse lungs post-APP infection (3). CD11b deficiency exacerbates methicillin-resistant *Staphylococcus aureus*-induced sepsis by upregulating the inflammatory response of macrophages (24). Our results showed that CD11b^−^Mø highly expressing TLR4 was also accumulated in the spleen of APP-infected mice, suggesting that CD11b^−^Mø plays an important anti-infective early role during APP infection.

Adaptive immune cells, such as T cells, especially memory T cells, have been reported to modulate innate immune cells in the early stages of infection (25). To date, studies on T-cell function in APP infections are scarce and have focused mainly on the chronic phase, as T cells are traditionally adaptive immune cells (26). In our study, the majority of the top six clusters contributing to the time-specific profile of the infection were T and B cells (70% of all top clusters) in the spleen. In addition, CD24^hi^ MHCII^+^CD8^+^ T_EM_ cells were significantly increased at 12 h and had a strong ability to secrete cytokines IL-10, IFN-γ, and IL-17A. This suggests that when pathogens invade the organism, these cells can rapidly release a large number of cytokines that act on immune cells to help the organism establish an effective immune response. In addition, the specific spatial localization of T cells determined their function. For example, in the epithelial layer of the small intestine, resident memory T cells mainly play the role of surveillance and early response, while in the lamina propria, they interact more with other immune cells to maintain immune homeostasis (27). This suggests that CD24^hi^MHCII^+^CD8^+^ T_EM_ cells may have specific spatial localization in different tissues and participate in immune responses through interactions with multiple cells. The role of T- and B-cell subpopulations in APP infection deserves further in-depth investigation. In addition, the changes in CD24^hi^ Ly-6C^+^ CD8^+^ T_EM_ cells in the spleen and inguinal lymph nodes following infection do not follow an identical trend, highlighting the tissue-specific nature of the immune response.

Because the dynamics of the immune components are consistent with the severity of the lung illness, the immunological status in the blood indicates the severity of the lung disease. This implies that immune cells in peripheral blood can be used to track the progression of an APP infection. After APP infection, we revealed unprecedented heterogeneity in the peripheral blood and, in addition to well-known key immune cells, identified several previously unidentified clusters such as (Mye-8, 19, 10, 14, 16), whose function deserves future determination. In addition, MHC-II^+^ PMN were present in the peripheral blood of mice in the absence of infection, suggesting the presence of PMN with antigen-presenting cell characteristics. After 24 h of APP infection, only CD24^hi^ PMN were significantly enriched and exhibited characteristics of apoptotic cells. This prompted apoptosis, avoided excessive inflammation, and ensured that the organism effectively cleared bacterial infections and maintained immune homeostasis. We also observed changes in the expression of functional molecules (e.g., CD11c, Ly-6C) on major T-cell subpopulations with the onset and progression of the disease, reflecting T-cell activation and remodeling in the peripheral blood during APP infection. Meanwhile, clustering analysis showed that the immune cell compositions of samples from 0 h and 24 h of infection were similar, and samples from the acute phase of infection (6 h and 12 h) were clustered with each other, indicating that the changes in peripheral blood immune cells were closely related to the period of APP infection. More importantly, these disease-specific changes can be fully captured via single-cell analyses.

Although we explored immune cell subpopulations in different organs after APP infection, the function of key cell subpopulations needs to be further investigated. In addition, we are well aware that it is crucial to understand the cellular interactions and functional status within each tissue. Therefore, our further research will focus on the spatial localization of key cell subpopulations and their interactions in each tissue after APP infection.

In conclusion, using high-dimensional mass cytometry, we have generated comprehensive immune response profiles and discovered key infection-specific clusters in the spleen and peripheral blood during APP infection. Therefore, our research not only provides a data set repository for the study of porcine contagious pleuropneumonia but also establishes the foundation for the development of new drugs and vaccines for APP infection from the aspect of targeting host immune responses.

## Data Availability

Mass cytometry data are available via Flow Repository (ID: FR-FCM-Z4SS) DOI: 10.1186/s13567-023-01207-4. The other materials are available from the corresponding author upon reasonable request.

## SUPPLEMENTAL MATERIAL

Supplemental material spectrum.02665-24-s0001.docx Fig. S1 to S5.

ASM does not own the copyrights to Supplemental Material that may be linked to, or accessed through, an article. The authors have granted ASM a non-exclusive, world-wide license to publish the Supplemental Material files. Please contact the corresponding author directly for reuse.
