# Supplementary material for: Mass cytometry analysis reveals a cross-tissue immune landscape in Actinobacillus pleuropneumoniae-induced pneumonia
Source: Microbiol Spectr. 2025 Apr 16;13(6):e02665-24. doi: 10.1128/spectrum.02665-24 (PMC12131827; doi:10.1128/spectrum.02665-24)
Supplement: Supplemental material — Fig. S1 to S5. [file spectrum.02665-24-s0001.docx]

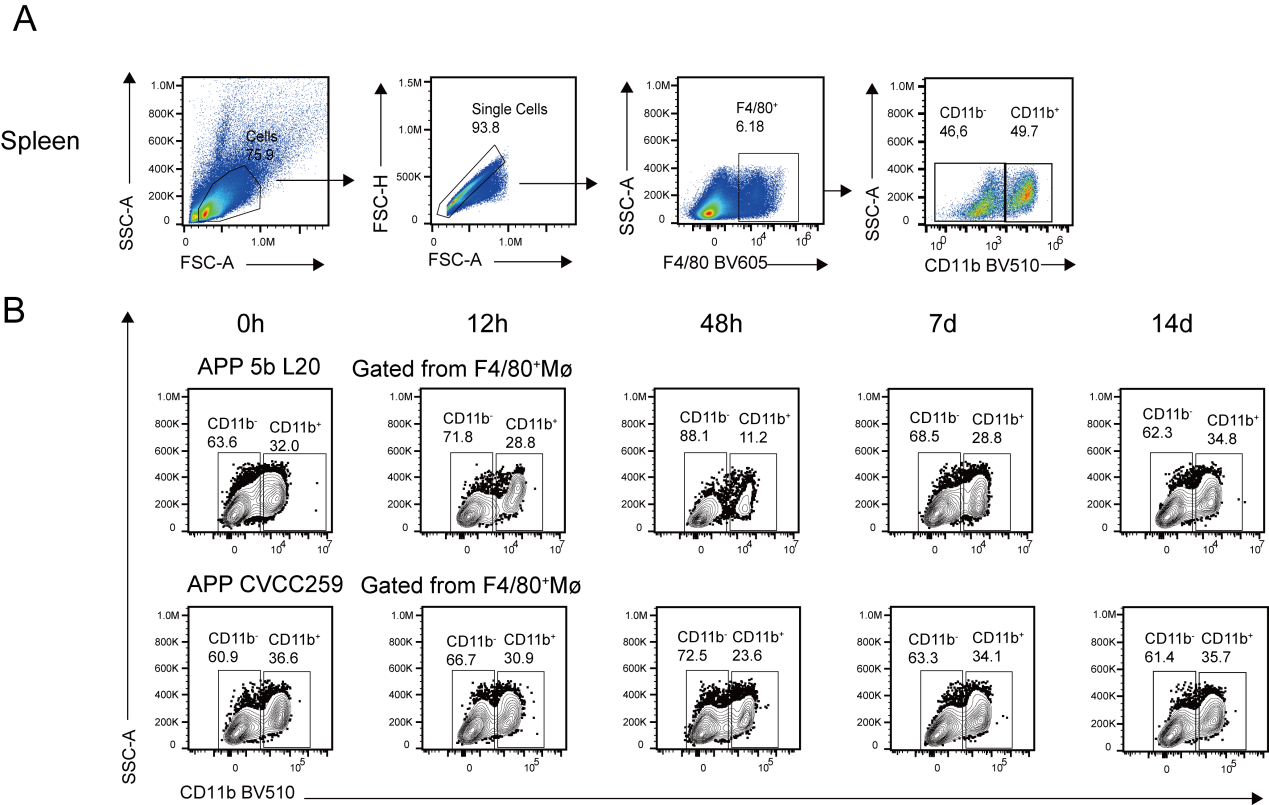


**Supplementary Fig. 1 Changes in the number of CD11b^+^ Mø, CD11b^-^ Mø after infection**

(A) Representative biaxial plots showing the gating strategy for CD11b^+^ Mø, CD11b^-^ Mø using flow cytometry. (B) Representative biaxial plots showing the cell frequencies of CD11b^+^ Mø, CD11b^-^ Mø **after infection with APP 5b L20 and APP CVCC259.**

**
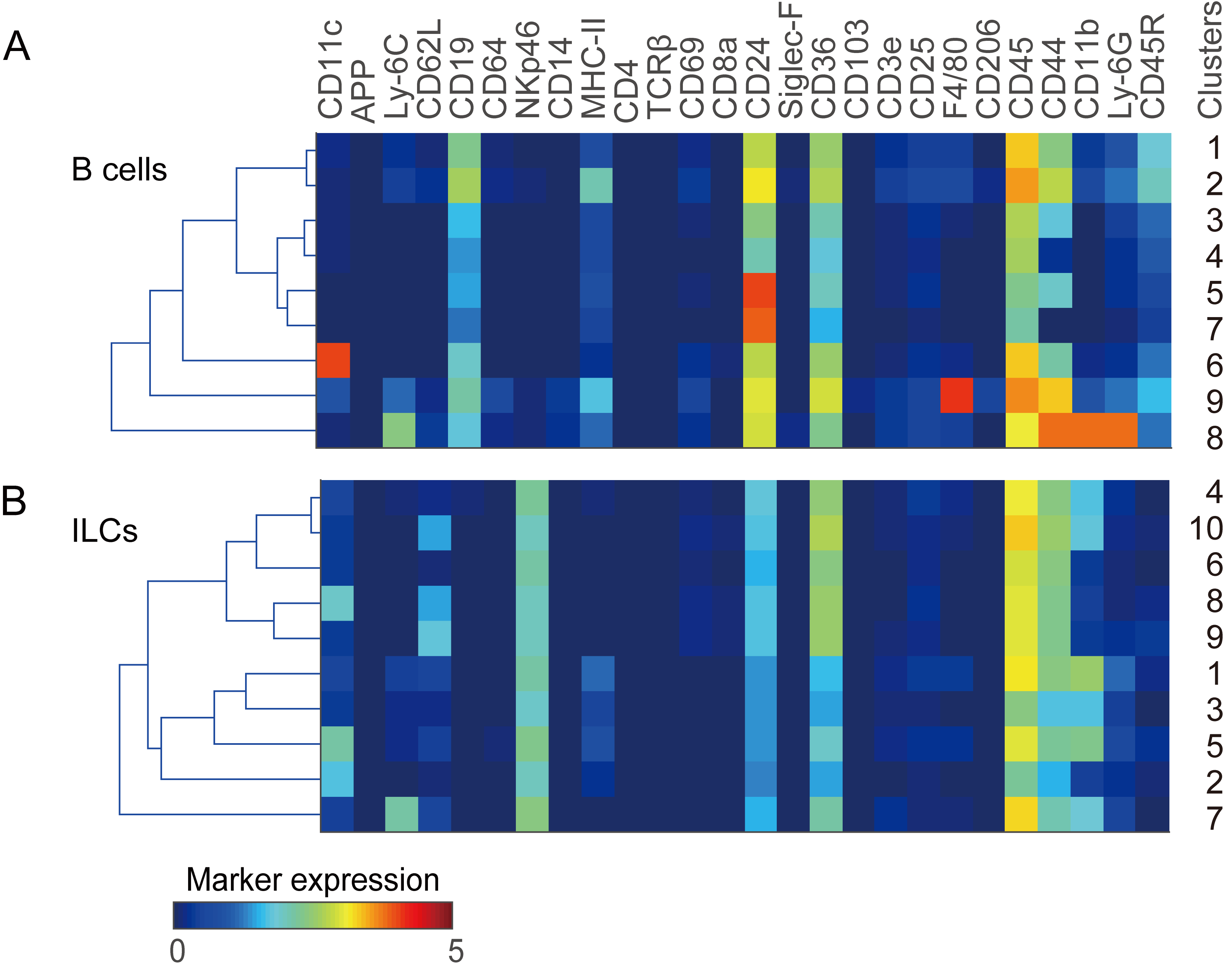
**

**Supplementary Fig. 2 Cluster identification of splenic B cell and ILC populations.**

1. B) Heatmaps display the median marker expression value and hierarchical clustering of the markers for clusters identified in each major immune lineage.

**
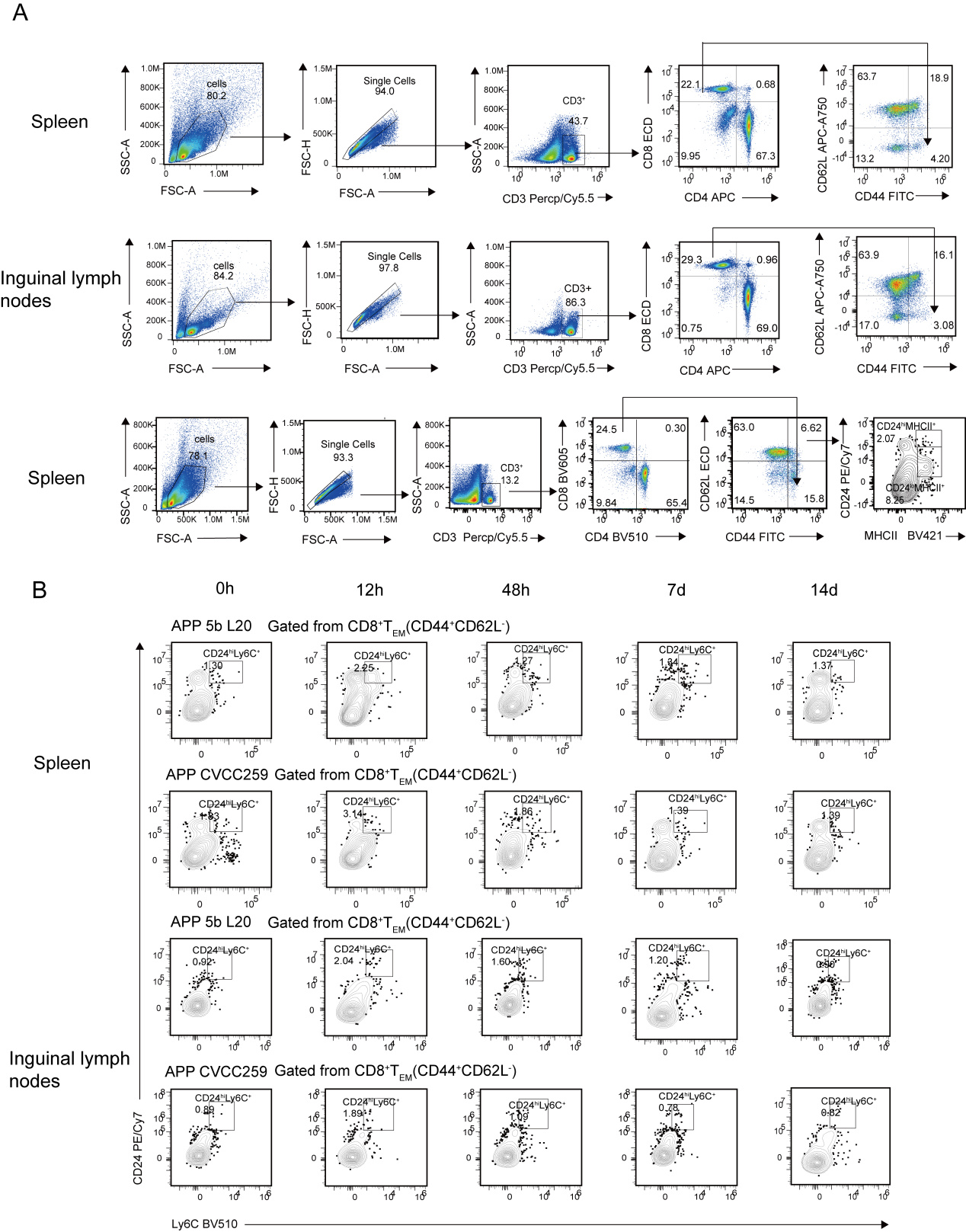
**

**Supplementary Fig. 3 Changes in the number of CD24^hi^Ly-6C^+^CD8^+^T_EM_ after infection**

1. Representative biaxial plots showing the gating strategy for CD24^hi^Ly-6C^+^CD8^+^T_EM,_ CD24^hi^MHCII^+^CD8^+^T_EM_ and CD24^lo^MHCII^+^CD8^+^T_EM_ using flow cytometry. (B) Representative biaxial plots showing the cell frequencies of CD24^hi^Ly-6C^+^CD8^+^T_EM_ after infection with APP 5b L20 and APP CVCC259.

**
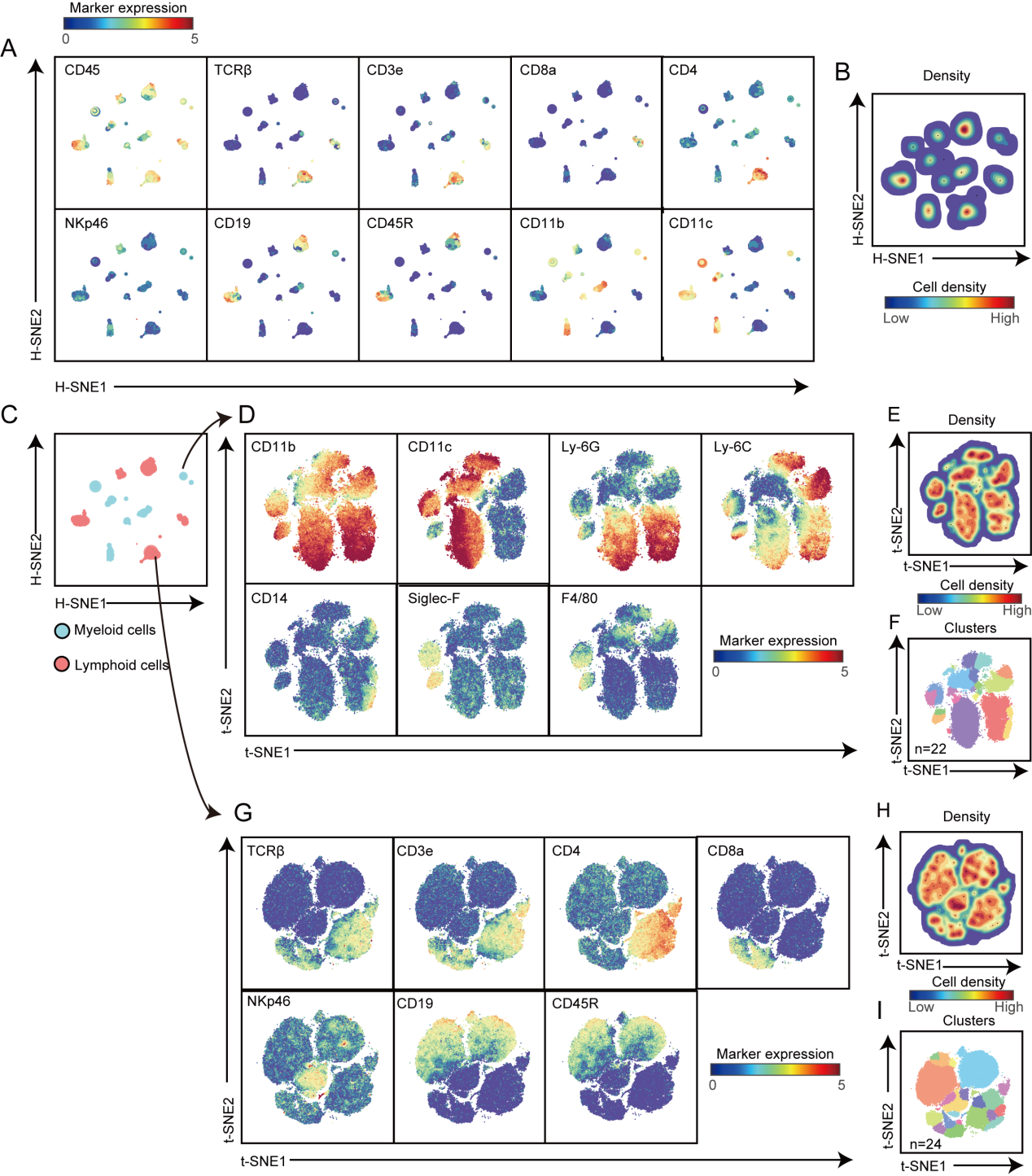
**

**Supplementary Fig. 4 Cluster identification in the lymphoid and myeloid cell compartment in PBMCs**

(A) HSNE embeddings of 157,951 immune cells derived from pooled peripheral blood (N=5). Each dot represents a landmark, whose size is proportional to the number of cells it represents. Colors indicate the ArcSinh5-transformed expression value of each indicated marker. (B) HSNE plots show the cell density. (C) A HSNE embedding of 157,951 immune cells derived from pooled peripheral blood (N=5). Colors represent different immune lineages. (D) t-SNE embeddings of myeloid cells show the ArcSinh5-transformed expression value of each indicated marker. (E) A density map shows the local probability density of the embedded cells. (F) A t-SNE plot shows cluster partitions. (G) t-SNE embeddings of lymphoid cells show the ArcSinh5-transformed expression value of each indicated marker. (H) A density map shows the local probability density of the embedded cells. (I) A t-SNE plot shows cluster partitions.

**
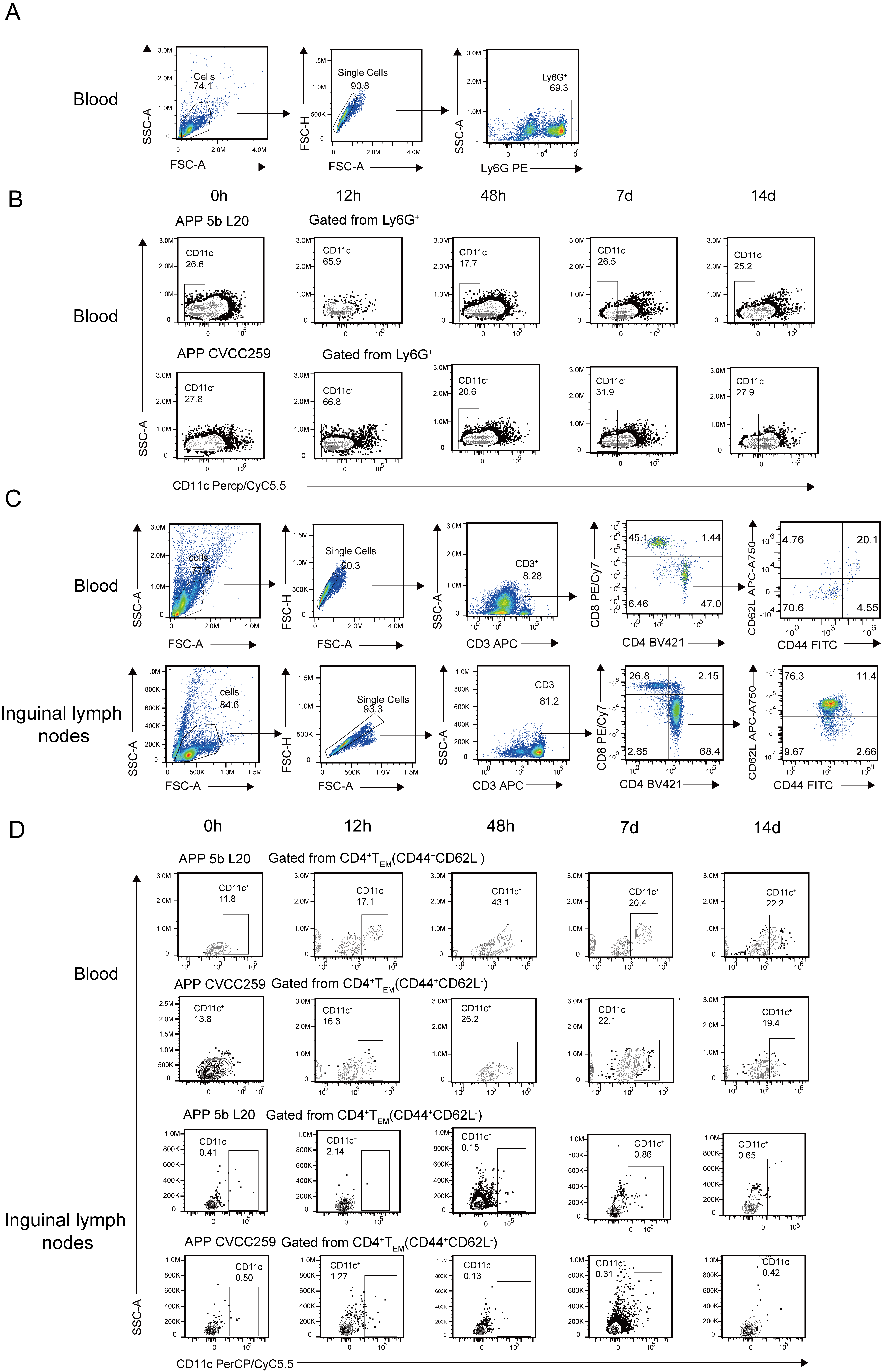
**

**Supplementary Fig. 5 Changes of the CD11c^-^PMN and CD11c^+^CD4^+^T_EM_ cells after APP infection**

(A) Representative biaxial plots showing the gating strategy for PMN using flow cytometry. (B) Representative biaxial plots showing the cell frequencies of CD11c^-^PMN **after infection with APP 5b L20 and APP CVCC259.** (C) Representative biaxial plots showing the gating strategy for **CD4^+^T_EM_** using flow cytometry. (D) Representative biaxial plots showing the cell frequencies of **CD11c^+^CD4^+^T_EM_ after infection with APP 5b L20 and APP CVCC259.**
